# The impact of external academic accreditation of undergraduate medical program on students’ satisfaction

**DOI:** 10.1186/s12909-021-03003-0

**Published:** 2021-11-09

**Authors:** Ayman Al-Eyadhy, Shuliweeh Alenezi

**Affiliations:** 1 Department of Pediatrics, College of Medicine, King Saud University, Riyadh, Saudi Arabia; 2 Department of Psychiatry, College of Medicine, King Saud University, Riyadh, Saudi Arabia; 3 Vice-Deanship of Quality and Development, College of Medicine, King Saud University, Riyadh, Saudi Arabia

**Keywords:** External accreditation, Students’ satisfaction, Quality improvement, Academic quality, Undergraduate medical program

## Abstract

**Background:**

The external academic accreditation is a quality assurance and auditing process that focuses on the structure, process, and outcome of the education. It is an interrupting and highly demanding process in terms of effort, time, financial, and human resources. However, it is unclear in the literature how much of these external quality assurance practices impeded in the accreditation processes would reflect on the other end of the learning pathway, including student satisfaction.

**Methods:**

A retrospective quantitative secondary data analysis, with a before-after comparison research design, was performed to evaluate external accreditation’s impact on students’ mean satisfaction score within two accreditation cycles at King Saud University (KSU)-Bachelor of Medicine, Bachelor of Surgery (MBBS) program.

**Results:**

The overall average students’ satisfaction scores pre-and-post the first accreditation cycle were 3.46/5 (±0.35), 3.71 (±0.39), respectively, with a *P*-value of < 0.001. The effect of post first accreditation cycle was sustainable for a couple of years, then maintained above the baseline of the pre-first accreditation cycle until the pre-second accreditation cycle. Similarly, the overall average students’ satisfaction scores pre-and-post the second accreditation cycles were 3.57/5 (±0.30) and 3.70 (±0.34), respectively, with a *P*-value of 0.04. Compared to the first accreditation cycle, the improvement of the mean score of students’ satisfaction rates was not sustained beyond the year corresponding to the post-second accreditation cycle.

**Conclusion:**

Both accreditation cycles were associated with an increased score in students’ satisfaction. The preparatory phase activities and navigation through the self-study assessment while challenging the program’s competencies are essential triggers for quality improvement practices associated with accreditation.

**Supplementary Information:**

The online version contains supplementary material available at 10.1186/s12909-021-03003-0.

## Introduction

Academic accreditation is a formal systematic external review typically mandated by the commissioning or regulatory bodies [1]. In Saudi Arabia, The National Center for Academic Accreditation and Evaluation (NCAAA) is responsible for accrediting all universities’ undergraduate programs [2]. This process consists of an in-depth evaluation of the program against sets of standards, covering its structure, processes, practices, procedures, and outcomes. The surveyors critically review the self-study report and then conduct on-site visits and interviews to clarify and verify the accreditation standards. The outcome of the accreditation process is either full or conditional or in the presence of significant concerns; the program is suspended until the implementation of appropriate corrective actions [3].

Effectiveness of accreditation as a tool of quality assurance requires active dynamics and positive integration between different program components [4–6]. For instance, meeting the accreditation standards at one point in time assumes the appropriate implementation of continuous improvement processes throughout the accreditation cycle. Therefore, structure and standards, surveyors’ experience, and positive perception of the program leadership are essential factors to reflect the positive impact of accreditation on the program [7–9]. Also, accreditation requires stakeholders’ true engagement and involvement of all program partners for effective and continuous improvement [10, 11]. Furthermore, accreditation has been reported to improve several program components, including the documentation, educational processes, and quality improvement practices [10, 12, 13].

On the other hand, accreditation has been criticized for its highly demanding financial and human resources [10, 14]. Moreover, and from different point of view, it is questioned for its short-term impact considering the latency period between two accreditation cycles. Therefore, despite the reported improvement in students’ performance, it is not clear how much of that is attributed to the accreditation given the complexity and varieties of the medical program activities [15].

Nevertheless, surveys’ effectiveness as a quality assurance tool requires optimization of the survey’s design, structure, contents, response rate, analysis, and action plan [16]. Collectively, addressing these points results in better surveys’ reliability, acceptability, and utility, comprising survey effectiveness cornerstones [17, 18]. For example, it is essential to have well-balanced perspectives on the survey themes, and equally important, not to include what might be beyond students’ perception, such as judging the course goals or validating its objectives [19]. The survey’s strength as an evaluation tool relies on a large pool of respondents and students’ engagement as stakeholders in the quality improvement cycle [19]. These students’ survey strengths unveil humanism and adult learning theories in the quality assurance process [20].

On the other side, the students’ satisfaction is an internal quality assurance process measuring students’ self-reported emotional reaction towards the educational process and outcome. The impact and reliability of student satisfaction have been debated in the literature [21]. Therefore, both of these two processes have been questioned for their impact as quality assurance tools. However, the use of self-report perception needs to be supported by evidence [22, 23]. Thus, students’ survey as a quality assurance tool has been a source of controversy and continues to be perceived skeptically by faculties [19]. On the other hand, limitations such as low response rate or student engagement, misinformed students’ expectations, and inherited misconception of faculties’ vulnerability for judgment need to be addressed appropriately [24, 25]. Overall, when balancing the role of student satisfaction survey and supporting it with qualitative tools like peer-review or focus group interviews, surveys are considered necessary formative tools for quality improvement [19].

This study highlights the use of both quality assurance tools to support the notion that they should be integrated longitudinally to perform a reliable quality improvement process beyond its role as a quality assurance tool at a one-time point. We examined the external accreditation process as an enforcing and validating tool for the students’ satisfaction internal process to address stakeholders’ concern who question students’ experience as an essential quality tool. Likewise, we used students’ experience as a reference point to address the concern of stakeholders questioning accreditation’s positive impact on students. Thus, this study’s research question is: what is the relation between the external accreditation process and the scores on students’ satisfaction surveys? Moreover, how do both of them get integrated into one longitudinal quality improvement model?

## Research methodology

### Hypothesis

**Fig. 1:**
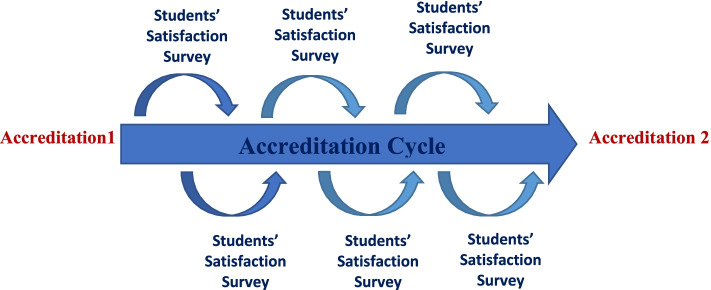
Relationship between accreditation cycles and students’ satisfaction

The authors tested the hypothesis that external accreditation would be associated with improved student satisfaction. Thus, both quality assurance tools, external accreditation, and student satisfaction surveys, should be incorporated into a longitudinal model of quality improvement, supporting the notion that both tools could support and complement each other (Fig. 1). This study’s outcome could highlight the indirect impact of accreditation on students’ satisfaction and add more insight into incorporating these tools into a comprehensive approach rather than using each one as a standalone tool.

### Study design

This study is a retrospective quantitative secondary data analysis with a before-after comparison research design.

### Study setting

This study utilized the data from the electronic records of King Saud University (KSU)-Bachelor of Medicine, Bachelor of Surgery (MBBS) program for the period of 10 academic years starting from 2009 to 2018. This covers two accreditation processes occurred; 2010 and 2016 performed by the NCAAA. However, the required practices and review process underwent minor updates during the second accreditation cycle.

### Participants/study population

KSU-MBBS program has 28 core courses divided into five year (levels) after a preparatory year, with average number of 1600 student in total. This study used the students’ satisfaction survey results as per the course evaluation questionnaire policy approved by the College of Medicine at KSU. The students’ satisfaction survey consists of 31 items corresponding to different theme such as course conduction, learning resources, practical/clinical experience, teaching staff, and assessments. The survey items are listed in Additional file 1. Inclusion criteria included all students’ satisfaction surveys for course evaluation at the end of each course conduction. Students’ satisfaction survey results for courses that were judged not to have an adequate response rate, less than 30% as per the college policy, were excluded.

### Sample size

A sample size of 320 student responses per course was determined to be adequate, based on the yearly average number of students in the program of 1600. However, to detect the difference in the mean score of students’ satisfaction for one-sample group before-and-after accreditation where the mean of course evaluations before accreditation = 3.5, SD ± 0.3, we assumed that the null hypothesis is true, one-sided alpha equal 5, and power equal 95%. Thus, the estimated sample size for the minimum number of program courses with adequate students’ satisfaction responses = 11 courses per academic year.

### Data collection methods

Data were retrieved through electronic records of King Saud University – College of Medicine, Academic Quality Unit. The self-study reports and related preparation documents of the first and second accreditation cycles were reviewed to understand, consider, and explore possible factors contributing to differences between both accreditation cycles. The score of pre-accreditation year indicates results of students’ satisfaction survey from the year preceding the accreditation, while the score of post-accreditation year indicates results of students’ satisfaction survey from the year following the accreditation. The year when the accreditation occurred where used as washout period.

### Data analysis

The outcome was measured by calculating the yearly mean score of students’ satisfaction of all eligible courses for each academic year and analyzing its changes over time pre- and-post accreditation processes. Themes’ analysis of the survey were compared, as well. These themes include course conduction, learning resources, practical/clinical experience, teaching staff, and assessment. The t-dependent test was used to detect the difference in the mean score pre- and-post accreditation. The level of statistical significance is considered when the *p*-value is less than 0.05. The effect size was estimated using Cohen’s d test and the interpretation scheme refers to effect sizes as small (d = 0.2), medium (d = 0.5), and large (d = 0.8) [26, 27].

### Ethical considerations

This study involves secondary data analysis without students’ identifier. However, the data is considered confidential data for the College of Medicine. All research documents will be secured and locked in the principal investigator’s office for two years post-publication as per institutional policy. The King Saud University Institutional Review Board (KSU-IRB) approved the proposal through the expedited track.

## Results

The students’ satisfaction of medical school undergraduate curriculum were included 28/28 (100%). The response rate was retrieved with an average overall response rate of 31.6%.

The descriptive statistics of overall students’ satisfaction over the consecutive 10 year during which two accreditation cycles occurred are shown in Table 1.

**Table 1:** Overall students’ satisfaction over 10 consecutive years during which two accreditation cycles occurred

		Cycle 1						Cycle 1		
	Pre 1	Acc 1	Post 1	Interim 1	Interim 2	Interim 3	Pre 2	Acc2	Post 2	Interim 4
	2009	2010	2011	2012	2013	2014	2015	2016	2017	2018
Courses n=	28	28	28	28	28	28	28	28	28	28
Mean	3.461	3.593	3.707	3.768	3.775	3.575	3.571	3.546	3.704	3.604
Std. Deviation	0.348	0.561	0.387	0.315	0.392	0.374	0.300	0.350	0.339	0.447
Minimum	2.900	2.200	3.000	2.900	3.100	2.800	3.000	2.600	2.900	2.400
Maximum	4.200	4.700	4.600	4.500	4.700	4.600	4.200	4.300	4.500	4.400

Pre 1: pre-accreditation 1st cycle, Acc 1: accreditation 1st cycle, Post 1: post-accreditation 1st cycle, Pre 2: pre-accreditation 2nd cycle, Acc 2: accreditation 2nd cycle, Post 2: post-accreditation 2nd cycle, interim: years med-way between accreditation cycles

### First accreditation cycle

The overall average students’ satisfaction scores pre-and-post the first accreditation cycle were 3.46/5 (±0.35), 3.71 (±0.39), respectively, with a *P*-value of < 0.001. The effect of post first accreditation cycle was sustainable for a couple of years, then maintained above the baseline of the pre-first accreditation cycle until the pre-second accreditation cycle. The effect size fell in the medium range of Cohen’s d test.

### Second accreditation cycle

**Fig. 2:**
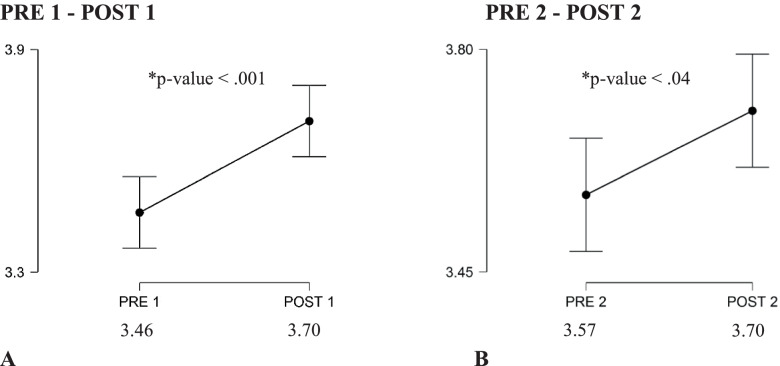
Paired t-test comparing mean students’ satisfaction before (PRE1) and after (POST1) 1st accreditation cycle, **A**; before (PRE2) and after (POST2) 2nd accreditation cycle, **B**

The overall average students’ satisfaction scores pre-and-post the second accreditation cycles were 3.57/5 (±0.30) and 3.70 (±0.34), respectively, with a *P*-value of 0.04. Compared to the first accreditation cycle, the improvement of mean score of students’ satisfaction rates was not sustained beyond the year corresponding to post-second accreditation cycle. The effect size fell in the small range of Cohen’s d test. The overall average students’ satisfaction scores pre-and-post accreditation cycles are demonstrated in the Fig. 2 below.

### The average students’ satisfaction score for survey themes’ analysis pre-and-post the accreditation cycles

These themes include course conduction, learning resources, practical/clinical, teaching staff performance, and assessment. The teaching staff evaluation scored the highest score (3.76/5) for survey themes’ analysis during the study period, followed by course conduction score (3.59/5).

### First accreditation cycle

The average students’ satisfaction scores pre-and-post the first accreditation cycle upon survey themes’ analysis revealed that difference was significant for course conduction and practical/clinical experience, while other themes included learning resources, teaching staff, and assessment showed improvement but not reaching statistical significance.

### Second accreditation cycle

The average students’ satisfaction scores pre-and-post the first accreditation cycle upon survey themes’ analysis revealed that difference was significant for course conduction, learning resources, practical/clinical experience, teaching staff, and assessment themes. The average students’ satisfaction score for survey themes’ analysis pre-and-post the accreditation cycles is demonstrated in Table 2 below.

**Table 2:** Comparison of different themes of students’ satisfaction Pre- and-Post accreditation cycles

Variable^a^	Accreditation 1					Accreditation 2				
	Pre	Post	t- test	***P*** value	Cohen’s d	Pre	Post	t- test	P value	Cohen’s d
Overall satisfaction, mean ± SD	3.46 ± 0.34	3.71 ± 0.39	−3.713	< .001	0.702	3.57 ± 0.30	3.70 ± 0.34	−2.156	0.040	0.407
Course conduction, mean ± SD	3.425 ± 0.372	3.757 ± 0.396	−3.299	0.003	0.624	3.550 ± 0.393	3.814 ± 0.365	−3.678	0.001	0.695
Practical/Clinical, mean ± SD	3.079 ± 0.487	3.575 ± 0.385	−4.542	< 0.001	0.858	3.471 ± 0.336	3.661 ± 0.446	−2.255	0.032	0.426
Learning Resources, mean ± SD	3.400 ± 0.361	3.614 ± 0.484	−1.952	0.061	0.369	3.536 ± 0.343	3.689 ± 0.359	−2.241	0.033	0.424
Teaching Staff, mean ± SD	3.679 ± 0.409	3.836 ± 0.343	−1.671	0.106	0.316	3.668 ± 0.294	3.889 ± 0.302	−4.136	< .001	0.782
Assessment, mean ± SD	3.40 ± 0.374	3.546 ± 0.441	−1.377	0.180	0.260	3.425 ± 0.387	3.625 ± 0.395	−2.510	0.018	0.474

^a^ The level of statistical significance is considered when the p-value is less than 0.05. The effect size was estimated using Cohen’s d test and the interpretation scheme refers to effect sizes as small (d = 0.2), medium (d = 0.5), and large (d = 0.8)

## Discussion

### The impact of external accreditation on the program’s quality

The journey of developing accreditation standards is continuous, where the focus on improving the quality of the program become holistic and multidimensional to include the acceptable national or international level of basic medical education, emphasis on the need for a student-centered curriculum, qualified teaching staff, healthy learning environment, and meeting the society’s needs with the ultimate objective of improving patient’s’ care [7, 28, 29]. This driving force towards assuring and improving medical education quality via external accreditation is further inspired by the World Federation of Medical Education’s (WFME) efforts for international standardization of medical education and recognition of accreditors; accreditation of accreditors [28, 29]. WFME triggered these international standardizations to guarantee an acceptable quality of medical education throughout the system of med­ical schools [29]. Despite these international efforts to standardize accreditation, the percentage of countries with an undergraduate program of medicine enforcing the national accreditation process remains sub-optimum while their processes vary widely [30].

The role of external accreditation in program improvement can be viewed from different perspectives; while the regulator or commissioning agencies to view it as an essential tool to meet the standards and assure a pre-set level of program quality, other stakeholders may view the accreditation process as a source of exhausting resources and efforts unfavorable balance for its cost-effectiveness. Although, accreditation may result in the improvement of the program’s administration and organization, its direct or indirect positive impact on students remains questionable [7, 10, 31–33]. The reason for this controversy is the paucity of research studies exploring such potential impact [34]. For instance, in a scoping review, Tackett et al.^36^ investigated the evidence base of medical school undergraduate program accreditation and found limited evidence to support existing medical school accreditation practices to guide the creation or improvement of accreditation systems. Only 30 cross-sectional or retrospective research were found [33]. Among their findings, the Middle East region is one of the areas with the least published research on medical school accreditation until 2019, which indicates the need for further evidence of accreditation’s impact on the undergraduate medical program in our region [33].

Moreover, upon further reflection, another reason for the paucity of research tackling this relation between accreditation and its impact on the undergraduate medical program is accreditation processes’ variable practice despite common themes of accreditation standards. Thus, this variability resulted in the lack of an agreed-upon framework for such research that can be adapted internationally with reasonable generalizability in different countries or regions [30]. Moreover, these different research viewpoints of accreditation on the undergraduate program led most publications reflecting the impact of accreditation to use a single indicator such as document analysis or participants’ perception regarding accreditation. For instance, linking accreditation with students’ performance in exams is a relatively widely adopted approach [15].

However, it inherits the limitation of such a cross-sectional and singular approach compared to an approach with a longitudinal (pre-post) assessment or approach that considers reproducibility over more than one accreditation cycle [2]. Blouin et al.^10^ sought to generate such a framework and explore potential indicators of accreditation effectiveness, value, and impact on medical education utilizing qualitative research design. They surveyed 13 Canadian medical schools who participated in national accreditation [35]. The study suggested general framework themes with direct impact and others with indirect impact. Theme with direct impact includes program processes, quality assurance, and continuous quality improvement program quality.

Furthermore, four other themes were considered indirect indicators of accreditation effectiveness, including student performance, stakeholder satisfaction, stakeholder expectations, and engagement. Therefore, considering this framework, our study focused on assessing scaled students’ satisfaction as an indirect measure of accreditation impact on medical programs. We also adapted pre- and post- longitudinal research design over two accreditation cycles, which is considered the most rigorous design of impact evaluation if experimental with-without comparison designs are not feasible [32, 36]. The before-after comparison is based on data collected at baseline (pre), intermediate (during), and after (post) the accreditation.

### Impact of accreditation on students’ satisfaction

In this study, both cycles were associated with an increased score of students’ satisfaction scale when considering the (pre-post) approach. Although the absolute difference between both scores might be perceived less meaningful, it is important to consider the context of the variability in students ‘scores, when not every student scored the average mean, which could help in understanding the scale of the change. Equally important to consider that the Likert scale is a calculated indices with no intrinsic meaning compared to an outcome with meaningful intrinsic values such as percentage of survival [26]. Therefore, we opted to provide the calculation of Cohen’s d test to demonstrate the meaningfulness and magnitude of change beyond the absolute difference and statistical significance [27]. The preparatory phase activities and navigation through the self-study assessment while challenging the program’s competencies are essential triggers for quality improvement practices associated with accreditation. The reinforcement of an internal quality improvement system is another major driving force to have a meaningful impact on accreditation [10, 28]. The difference in the sustained improvement post accreditation in both cycles is interesting. While improvement in the students’ satisfaction sustained longer post the first cycle, it was not apparent in the second cycle. However, the short follow up of one year post second cycle compared to 3 years follow up post first cycle makes it difficult and relatively premature to interpret such findings. The themes’ analysis of the survey revealed interesting results. The positive impact of accreditation on students’ satisfaction in course conduction and practical/clinical experience was evident and reproducible over both cycles. Thus, our study reinforces the early study by Al Mohaimeed et al.^12^ following their first cycle of the NCAAA accreditation, which described a positive experience with accreditation in educational processes, administration, and curriculum implementation.

Moreover, in our study, the second cycle was associated with a significant impact on most of the survey themes compared to the first cycle. Upon reviewing the self-reported study of both accreditation cycles, this could be related to restructuring some of the college’s facilities and significant enhancement of students’ support services and temporal relationship with college building expansion during the second accreditation. Also, the review of the self-study report and preparation documents revealed that the second cycle was accompanied by higher engagement of teaching staff by creating departmental and college-wide permanent committees focusing on academic quality and fostering continuous development. Another interesting aspect of the students’ satisfaction association with accreditation in this study is the sustainable high satisfaction related to teaching staff performance over the study period, which was statistically significant and carried the highest effect size during the second cycle. Although this could be multifactorial, the teaching staff’s engagement during the preparation process, which may run over an average of two to three years, could play an essential role in this aspect. Furthermore, a broader scope of awareness and preparation campaigns among teaching staff were carried on during the second cycle to emphasize the culture of academic quality improvement. The teaching staff’s perspective and reframing of external accreditation result in higher acceptability of the accreditation as an ongoing improvement tool and strengthening the internal quality improvement system. In a recent qualitative study following the NCAAA accreditation cycle, Alrebish et al.^2^ elicited an essential theme of accreditation experience related to the perspective towards accreditation and its impact on the sustainability of quality improvement in undergraduate medical education. For instance, the perspective of accreditation as an external audit, and whether the program would pass the exam or not, is less likely to result in a sustainable positive impact on internal quality improvement practices [2].

### Viability and utilization of students’ satisfaction as a quality tool

There is no doubt that the utilization of student satisfaction as a quality improvement tool is widely debatable. This ongoing debate resulted in significant research on students’ evaluation of teaching with a history dating back to the 1950s until recently [16, 18, 25, 37–44]. For instance, a special volume of New Directions for Institutional Research, which was devoted to this debate, suggested the preponderance of evidence towards the validity of students’ evaluation of teaching [38, 40, 42]. Many factors contribute to this controversy that follows the pendulum movement towards underrating and overeating its validity. From faculty point of view, the student satisfaction might be criticized for the following: variable students’ attitude, confounding effect of students’ performance, low response rate, reliability, and validity of survey as an evaluation tool for instruction, vulnerability to recall bias or bias due to instructor’s gender, personality, ethnic background, technical aspects of data collection, analysis, and construction of survey items [39, 45–48].

Moreover, along with the negative perception of students’ evaluation, students may view the survey as a futile effort and burden rather than a way to improve the course instruction, particularly when the quality improvement loop is not closed appropriately. On the flip side, authorities tend to overrate the students’ evaluation and view them as a truly objective measure; they may use it alone or with others as a summative assessment, rather than a formative tool, for course instruction and decisions related to instructors’ hiring or promotion [16]. These misperceptions about students’ satisfaction by different stakeholders are likely the result of misuse or misinterpretation of students’ satisfaction. Not only that, these misperceptions could trigger a vicious circle of mistrust and resistance among program stakeholders. For instance, faculties tend to resist the notion of students being empowered to evaluate the faculty, while reframing the student evaluation to be a type of formative input or feedback to improve the students’ experience and course instruction can lead to higher acceptance among teaching staff. Similarly, the misinterpretation by authorities of student satisfaction and using it as a surrogate marker for learning effectiveness during course evaluation needs to be reframed by separating the two issues and realizing that student learning does not equal student experience or satisfaction [35].

The notion that student learning does not equal student satisfaction should not undermine the student’s experience and its vital role in the learning environment. Both need to complement each other, students will be more satisfied when they learn better, and they will learn more if they are highly satisfied. There is currently more emphasis on keeping end-user needs or customer experience at the center of every professional business model or accreditation [29]. To summarize the result of this debate about students’ evaluation and satisfaction, it is clear that student evaluation is currently and likely will remain an essential component of teaching and learning quality improvement. However, the appropriate interpretation and wise use are of paramount importance for its positive impact. In this study, we found a clear association between the timing of accreditation and an increase in student satisfaction scores when comparing pre-and-post accreditation. It demonstrates that accreditation has positively impacted the students’ satisfaction with this range of 10-year data. Although this positive correlation remains difficult to be labeled as a causality effect, the evident temporal relationship during two cycles suggests a clear direct or indirect impact of accreditation on the students’ satisfaction. This impact on students’ satisfaction highlights a very interesting aspect of accreditation’s impact on the medical program. It reflects the self-reported perception of the emotional dimension and its interaction within the learning environment, which is not easily measured otherwise infrequently considered or encountered in longitudinal accreditation research [34].

This study also illustrates that there was a drop in the scores of students’ satisfaction in-between accreditation cycles. Although this drop was relative and could be within an acceptable range, it illustrates the difficulty in maintaining the momentum associated with accreditation. Thus, there is a need to enhance the continuous internal quality improvement system to fill in this gap and bridge accreditation consecutive cycles together. Adapting student satisfaction as an essential component of this internal quality improvement system can play an important role in developing a timely and well-integrated quality system that can longitudinally sustain program improvement. The relatively small magnitude and narrow range of change in students’ satisfaction scores over the study period should be interpreted with caution, as each year’s average value reflects the average of a large pool of students’ responses to all program courses.

### Strengths and limitations

One of the strengths of this study is being responsive to the needs verbalized within the medical education community nationally and in the international literature to answer an important question [34, 35]. The outcome measured is hypothesis-driven and in accordance with the previously proposed research framework to explore the accreditation impact. The design of pre-and-post intervention analysis, and longitudinal data collection over a range of 10 years, to cover the range of two cycles of accreditation, are of added value to this study. Although the data included is large, being from a single institution is a relative limitation. This study’s generalizability may also be considered with caution, given the national perspective of the NCAAA accreditation standard and the potential effect of cultural differences related to students’ satisfaction.

## Supplementary Information

**Additional file 1.** Course Evaluation Survey.

## Data Availability

The data that support the findings of this study are available from King Saud College of Medicine but restrictions apply to the availability of these data, which were used under license for the current study, and so are not publicly available. Data are however available from the authors upon reasonable request and with permission of King Saud College of Medicine.

## Abbreviations

NCAAA: National Center for Academic Accreditation and Evaluation
MBBS: Bachelor of Medicine, Bachelor of Surgery
KSU: King Saud University
KSU-IRB: King Saud university Institutional Review Board
WFME: World Federation of Medical Education’s
