# Correction to: The impact of external academic accreditation of undergraduate medical program on students’ satisfaction

**DOI:** 10.1186/s12909-021-03028-5

**Published:** 2022-01-28

**Authors:** Ayman Al-Eyadhy, Shuliweeh Alenezi

**Affiliations:** 1 Department of Pediatrics, College of Medicine, King Saud University, Riyadh, Saudi Arabia; 2 Department of Psychiatry, College of Medicine, King Saud University, Riyadh, Saudi Arabia; 3 Vice-Deanship of Quality and Development, College of Medicine, King Saud University, Riyadh, Saudi Arabia

**Correction to: BMC Medical Education (2021) 21:565**

**https://doi.org/10.1186/s12909-021-03003-0**

Following publication of the original article [1], we have been informed that author Shuliweeh Alenezi should be affiliated as follows:

Department of Psychiatry, College of Medicine, King Saud University, Riyadh, Saudi Arabia.

Vice-Deanship of Quality and Development, College of Medicine, King Saud University, Riyadh, Saudi Arabia.

The original article has been corrected.
