# Supplementary material for: The impact of external academic accreditation of undergraduate medical program on students’ satisfaction
Source: BMC Med Educ. 2021 Nov 9;21:565. doi: 10.1186/s12909-021-03003-0 (PMC8576880; doi:10.1186/s12909-021-03003-0)
Supplement: Supplementary file 1 — Additional file 1. Course Evaluation Survey. [file 12909_2021_3003_MOESM1_ESM.docx]

**Appendix A:**

Course Evaluation Survey

| Course organization and planning | 1- The course objectives are clear to the student from the start of the course. |
| --- | --- |
|  | 2- The conduct of the course was consistent with the course outline. |
|  | 3- I was told about requirements to pass this course. |
| Learning resources | 1- The learning sources has been clarified at the beginning of the course. |
|  | 2- The learning resources were appropriate and useful. |
|  | 3- The learning resources were available. |
| Faculty staff experiences | 1- The teaching staff are punctual in terms of the educational activities dates and times. |
|  | 2- Teaching staff of this course are excellent. |
|  | 3- I can find the teaching staff outside lecture times for questions and discussions. |
|  | 4- Faculty staff respect the students and we have good relation with them. |
|  | 5- Faculty staff encourage the students to ask questions and to start discussions. |
| Practical sessions satisfaction | 1- Practical sessions were complementary to the course and very useful. |
|  | 2- Frequency of practical / clinical sessions is appropriate. |
|  | 3- The variety of practical / clinical material is reasonable. |
|  | 4- The practical / clinical instructor interaction and supervision is satisfactory. |
|  | 5- The hands-on experience & skill learning are useful. |
| Overall course satisfaction | 1- The effort and time I spent in this course was reasonable. |
|  | 2- I feel that this course will make me a better doctor. |
|  | 3- I am happy with this course in general. |
| Course assessment | 1-The assessment methods has been clarified at the beginning of the course |
|  | 2 -The overall assessment methods for this course is appropriate |
|  | 3 -Exam questions were matched with course/curriculum objectives |
